# Characterization, Genetic Analyses, and Identification of QTLs Conferring Metabolic Resistance to a 4-Hydroxyphenylpyruvate Dioxygenase Inhibitor in Sorghum (*Sorghum bicolor*)

**DOI:** 10.3389/fpls.2020.596581

**Published:** 2020-12-09

**Authors:** Balaji Aravindhan Pandian, Aruna Varanasi, Amaranatha R. Vennapusa, Rajendran Sathishraj, Guifang Lin, Mingxia Zhao, Madison Tunnell, Tesfaye Tesso, Sanzhen Liu, P. V. Vara Prasad, Mithila Jugulam

**Affiliations:** ^1^Department of Agronomy, Kansas State University, Manhattan, KS, United States; ^2^Bayer Crop Science, St. Louis, MO, United States; ^3^Department of Plant Pathology, Kansas State University, Manhattan, KS, United States; ^4^Sustainable Intensification Innovation Lab, Kansas State University, Manhattan, KS, United States

**Keywords:** *Sorghum bicolor*, resistance, QTL mapping, single gene inheritance, tembotrione

## Abstract

Postemergence grass weed control continues to be a major challenge in grain sorghum [*Sorghum bicolor* (L.) Moench], primarily due to lack of herbicide options registered for use in this crop. The development of herbicide-resistant sorghum technology to facilitate broad-spectrum postemergence weed control can be an economical and viable solution. The 4-hydroxyphenylpyruvate dioxygenase-inhibitor herbicides (e.g., mesotrione or tembotrione) can control a broad spectrum of weeds including grasses, which, however, are not registered for postemergence application in sorghum due to crop injury. In this study, we identified two tembotrione-resistant sorghum genotypes (G-200, G-350) and one susceptible genotype (S-1) by screening 317 sorghum lines from a sorghum association panel (SAP). These tembotrione-resistant and tembotrione-susceptible genotypes were evaluated in a tembotrione dose–response [0, 5.75, 11.5, 23, 46, 92 (label recommended dose), 184, 368, and 736 g ai ha^–1^] assay. Compared with S-1, the genotypes G-200 and G-350 exhibited 10- and seven fold more resistance to tembotrione, respectively. To understand the inheritance of tembotrione-resistant trait, crosses were performed using S-1 and G-200 or G-350 to generate F_1_ and F_2_ progeny. The F_1_ and F_2_ progeny were assessed for their response to tembotrione treatment. Genetic analyses of the F_1_ and F_2_ progeny demonstrated that the tembotrione resistance in G-200 and G-350 is a partially dominant polygenic trait. Furthermore, cytochrome P450 (CYP)-inhibitor assay using malathion and piperonyl butoxide suggested possible CYP-mediated metabolism of tembotrione in G-200 and G-350. Genotype-by-sequencing based quantitative trait loci (QTL) mapping revealed QTLs associated with tembotrione resistance in G-200 and G-350 genotypes. Overall, the genotypes G-200 and G-350 confer a high level of metabolic resistance to tembotrione and controlled by a polygenic trait. There is an enormous potential to introgress the tembotrione resistance into breeding lines to develop agronomically desirable sorghum hybrids.

## Introduction

Grain sorghum [*Sorghum bicolor* (L.) Moench ssp. *bicolor*] is one of the most versatile crops with multiple uses, including for food, feed, and fuel (Ciampitti and Prasad, 2019). Sorghum performs better than corn (*Zea mays*) under rainfed and low input conditions (Valadabad et al., 2000; Staggenborg et al., 2008). The US is the largest producer of grain sorghum in the world, and almost half of the US grain sorghum is produced in Kansas (USDA-NASS, 2020). Sorghum is primarily grown for cattle feed and ethanol production in the US, whereas it is a staple food for millions of people in Africa, India, and South America (Taylor et al., 2006; Dahlberg et al., 2012). Weed infestation, specifically grass weed species, pose a major problem in sorghum production and can reduce the crop yields up to 60% if left uncontrolled (Thompson et al., 2019; Dille et al., 2020). Palmer amaranth (*Amaranthus palmeri*), common waterhemp (*Amaranthus tuberculatus*), kochia (*Bassia scoparia*), common ragweed (*Ambrosia artemisiifolia*), and common lambsquarters (*Chenopodium album*) are the major broadleaf weeds and johnsongrass (*Sorghum halepense*), shattercane (*Sorghum bicolor* ssp. *verticilliflorum*), and large crabgrass (*Digitaria sanguinalis*) are major grass weeds found in grain sorghum fields (Stahlman et al., 2000; Smith et al., 2010). A wide range of postemergence (POST) herbicides are available to control broad-leaved weeds in sorghum. However, herbicide options for POST control of grasses are limited due to the susceptibility of sorghum to commonly used grass control herbicides (Thompson et al., 2019).

The 4-hydroxyphenylpyruvate dioxygenase (HPPD) inhibitors (e.g., mesotrione or tembotrione) are widely used to control a broad spectrum of weeds including grasses in corn because it can effectively metabolize HPPD inhibitors (Williams and Pataky, 2010). However, these herbicides are not registered as POST in sorghum due to crop injury. Although these herbicides are widely used, to date only two weed species, i.e., Palmer amaranth and common waterhemp, have been documented to have evolved resistance to HPPD inhibitors (Heap, 2020). These herbicides inhibit the HPPD enzyme, which is important for the conversion of 4-hydroxyphenyl pyruvate to homogentisate, an intermediate in plastoquinone and tocopherol biosynthesis pathway in plants (Lee et al., 1998). Plastoquinone is essential for the carotenoid biosynthesis, which protects the chlorophyll by absorbing excited electrons released during photosynthesis. Depletion of carotenoids causes damage to the chlorophyll by photo-oxidation resulting in bleaching followed by necrosis and plant death (Dankov et al., 2009). HPPD inhibitors include four chemical families isoxazole, pyrazole, pyrazolone, and triketones, and were introduced in the 1980s for weed control (van Almsick, 2009).

Herbicide resistance in plants can be conferred by two major mechanisms: (1) target-site resistance (TSR): mutation(s) in the herbicide target gene leading to the reduced affinity of the target enzyme for herbicide binding or due to increased expression of target enzyme; (2) non-target site resistance (NTSR): increased metabolism or reduced absorption/translocation of herbicides (Gaines et al., 2020). Metabolism of HPPD inhibitors by cytochrome P450 enzyme (CYPs) activity is the most common mechanism of resistance found in crops as well as weeds (Ahrens et al., 2013). Nonetheless, increased expression of *HPPD* gene has also been reported in some biotypes of Palmer amaranth (Nakka et al., 2017). Recently, a modified *HPPD* gene from *Pseudomonas fluorescens* and *Avena sativa* which is insensitive to HPPD inhibitors was used to develop transgenic soybeans (*Glycine max*) resistant to HPPD inhibitors by Bayer Crop Science (Matringe et al., 2005; Dreesen et al., 2018) and Syngenta (Hawkes et al., 2016), respectively. Dupont-Pioneer used an insensitive shuffled variant of corn *HPPD* gene that confers a high level of resistance to HPPD inhibitors in soybean (Siehl et al., 2014).

CYPs are one of the largest enzyme families involved in xenobiotic metabolism in microorganisms, insects, plants, and humans imparting resistance, respectively, to antibiotics, insecticide, herbicide, and drugs (Pandian et al., 2020). The activity of CYPs can be inhibited using several chemical compounds: 1-aminobenzo-triazole (ABT), tetcyclacis (TET), piperonyl butoxide (PBO), tridiphane, and organophosphate insecticides such as malathion and phorate (Siminszky, 2006; Busi et al., 2017). Treatment with CYP inhibitors before herbicide application will competitively reduce the CYP activity resulting in decreased metabolism of herbicide, thereby reducing the level of resistance (Siminszky, 2006). CYP inhibitors have been widely used to determine metabolic resistance to herbicides in several plant species.

Specifically, malathion and PBO were used to demonstrate the inhibition of CYP activity and the reversal of crop tolerance to HPPD inhibitors in corn (Ma et al., 2013; Oliveira et al., 2018).

Development of sorghum hybrids resistant to HPPD inhibitors will provide POST herbicide options to control grass weeds (Thompson et al., 2019). Tembotrione is a triketone herbicide which has broad-spectrum activity including grass weeds. Furthermore, the efficacy of tembotrione is high on grass weeds compared with other triketones (Ahrens et al., 2013). Mesotrione, a triketone herbicide similar to tembotrione, is registered for pre-emergence (PRE) use in sorghum but not as POST; however, tembotrione is not registered for PRE or POST usage in sorghum. We have used sorghum association panel (SAP) composed of homozygous sorghum genotypes representing all cultivated races from diverse geographic regions including widely used US breeding lines. We hypothesize that screening diverse genotypes from the SAP will facilitate the identification of genotypes resistant to tembotrione; such resistance, similar to maize, is associated with CYP-mediated metabolism. The specific objectives of this research were to identify and characterize sorghum genotypes with resistance to tembotrione, to investigate the inheritance and mechanism of resistance to tembotrione, and to identify genetic loci conferring tembotrione resistance.

## Materials and Methods

### Plant Materials

Sorghum genotypes from the SAP (Casa et al., 2008) were used in this study. A commercial sorghum hybrid Pioneer 84G62 and a corn inbred B73 (naturally resistant to tembotrione) were also used for comparison.

### *In vitro* Screening

Sorghum genotypes (∼317) from SAP along with Pioneer 84G62 and B73 were used for initial screening with tembotrione under *in vitro* conditions. Seeds of all genotypes were germinated in plastic Petri dishes (100 mm diameter × 20 mm height) containing 0.8% w/v solidified agar medium (PhytoTech Laboratories, Lenexa, KS, United States). Seeds were surface sterilized with 2% ethanol for 2 min followed by 5% (v/v) sodium hypochlorite for 15 min. Subsequently, seeds were rinsed two to three times with sterile distilled water before placing them on the agar medium. About 8–10 seeds were placed in each Petri dish for germination and incubated in a growth chamber maintained at 24°C with 16/8 h (day/night) photoperiod under a photosynthetic flux of 200 μmol m^–2^ s^–1^ (daylight fluorescent tubes). On germination, seedlings at three-leaf stage were transferred to culture vessels (PhytoTech Laboratories) containing solidified agar supplemented with 0.25 μM molecular grade tembotrione (Sigma-Aldrich, St. Louis, MO, United States). All transplanted culture vessels were incubated in the same growth chamber, maintained at the same conditions as indicated previously. The experiment was conducted with four to eight biological replicates (two culture vessels with two to four plants in each culture vessel). The response of genotypes to tembotrione treatment was evaluated visually (percent injury) at 2 and 4 weeks after treatment (WAT) based on a 0–100% rating scale (0% is no injury and 100% is complete death) (Abit et al., 2009).

### Whole Plant Assay

Ten sorghum genotypes (Supplementary Table S1) that exhibited minimum injury and S-1 that was found highly susceptible to tembotrione under *in vitro* conditions were tested along with Pioneer 84G62 for their response to tembotrione under greenhouse conditions. The seeds of sorghum genotypes were planted in square pots (15 × 15 × 15 cm) filled with a potting mixture (ProMix Ultimate; Premier Tech Horticulture, Mississauga, Ontario, Canada). The seedlings at three-leaf stage (Roozeboom and Prasad, 2019) were transplanted in square pots (6 × 6 × 6 cm) and grown in a greenhouse maintained at 25/20°C, 15/9 h day/night photoperiod with a photosynthetic photon flux density of 750 μmol m^–2^ s^–1^ and relative humidity of 60 ± 10%. The plants were fertilized (Miracle GRO^®^ all-purpose plant food; ScottsMiracle-Gro, Marysville, OH, United States) as needed. The sorghum seedlings at five-leaf stage (Roozeboom and Prasad, 2019) were treated with tembotrione (Laudis; Bayer Crop Science, St. Louis, MO, United States) at 92 g ai ha^–1^ (field recommended dose) with 0.25% methylated soy oil (Destiny; WinField United) using a bench-top track spray chamber (Generation III; De Vries Manufacturing, Hollandale, MN, United States) equipped with a single flat-fan nozzle (80015LP TeeJet tip; Spraying Systems, Wheaton, IL, United States) delivering 187 L ha^–1^. Each plant was considered as an experimental unit, eight replications were used for each genotype. The response of sorghum genotypes to tembotrione treatment was evaluated by visual injury rating as described previously (Abit et al., 2009). The above-ground plant biomass was harvested 3 WAT and dried in an oven at 60°C for 72 h. The weight of dried biomass was recorded as described later in a separate section. The experiment was repeated two times following the same procedure and growth conditions.

### Dose-Response Assay

Two genotypes, i.e., G-200 and G-350, that exhibited the least injury, and one highly susceptible genotype S-1 that exhibited the highest injury to tembotrione treatment identified from *in vitro* and whole plant assays, along with Pioneer 84G62 were tested in a tembotrione dose-response study to determine the level of resistance. The sorghum genotypes were treated with tembotrione at 0, 5.75, 11.5, 23, 46, 92, 184, 368, and 736 g ai ha^–1^. The experiment was conducted following the same plant growth conditions and herbicide application procedure as described previously in the whole plant assay. The experiment was conducted in a completely randomized design with four replications and repeated twice. The above-ground plant biomass reduction was measured as described previously.

### Field Testing

On confirmation of the level of resistance to tembotrione in the greenhouse, the tembotrione-resistant sorghum genotypes G-200 and G-350 were evaluated in comparison with S-1 (susceptible) and Pioneer 84G62 (commercial hybrid) under field conditions. Experiments were conducted in summer of 2017 at two KSU research sites: Ashland Bottoms Research Farm, Manhattan (Reading silt loam soil type; Pachic Agriustolls taxonomic class); and Agricultural Research Center, Hays (Harney silt loam soil type; Typic Agriustolls taxonomic class). *S*-Metolachlor at 2 kg ai ha^–1^ was applied as a pre-emergence herbicide to all plots at both sites to suppress existing weeds in the field before planting sorghum. Seeds of sorghum genotypes were planted in both locations on June 6, 2017 with a 76-cm space between rows and 7.6-cm space between plants, and 2.5 cm planting depth at a rate of 172,000 seeds ha^–1^ (Abit et al., 2011). The experimental plots were 3 m wide and 6 m long with four rows; the resistant or susceptible genotypes were planted in the middle two rows along with two border rows planted with Pioneer 84G62 to avoid herbicide drift from nearby treatments. POST application of tembotrione was made to individual plots when the sorghum plants reached five-leaf stage (Roozeboom and Prasad, 2019). Tembotrione treatments included 0, 92, 184, and 368 g ai ha^–1^. Herbicides were applied using a CO_2_-powered backpack-type research sprayer equipped with TurboTee 11002 nozzles calibrated to deliver 140 L ha^–1^ at 234 kPa. Experiments were conducted in a randomized complete block design with factorial arrangement with sorghum genotype and herbicide dose as the two factors. All treatments were replicated four times at each site. Sorghum response to herbicide treatments was visually assessed 1, 2, 4, and 8 WAT using a scale of 0 (no visible injury) to 100% (plant death) compared with the non-treated plants. At physiological maturity, grain weight was measured for each genotype. Three sorghum heads from each replication were randomly collected for each genotype separately from all treatments and dried in an oven at 60°C for 1 week. The dried sorghum heads from each plant were subsequently threshed to determine grain yield from a single plant.

### Generation and Evaluation of F_1_ and F_2_ Progeny

To study the inheritance and mapping of tembotrione resistance, direct and reciprocal crosses were performed using tembotrione-resistant (G-200 and G-350) and tembotrione-susceptible (S-1) genotypes in a crossing nursery at KSU research farm, Ashland Bottoms, KS. The crosses were made using the plastic bag method (Rakshit and Bellundagi, 2019). The F_1_ seeds were harvested from individual plants. The F_1_ progeny from S-1 × G-200 and S-1 × G-350 were evaluated in a tembotrione dose-response assay by treating the plants with 0, 23, 46, 92, 184, and 368 g ai ha^–1^ of tembotrione. A total of 10–12 F_1_ plants from each cross (S-1 × 200 and S-1 × 350) per dose were treated and the true F_1_ plants were differentiated from the selfed plants by their response to tembotrione; hence, the susceptible (S-1) was used as female parent, and the plants that were derived by selfing would be killed at field recommended dose or higher. In addition, the selfed plants that survived at low doses were identified by parental phenotype and vigor and discarded.

Each plant was considered as an experimental unit with eight replications per dose. The same procedure, as described previously, was followed for tembotrione dose-response assay of F_1_ progeny. Three F_1_ plants per cross that exhibited resistance to tembotrione were selected to generate F_2_ seeds by self-pollination.

The F_2_ progeny were evaluated under greenhouse conditions with a single dose of tembotrione to determine the segregation of resistant and susceptible plants. Approximately 150 seedlings from a single F_2_ family (total of two F_2_ families) along with the parents were raised in the greenhouse (as described previously under the same growth conditions). The seedlings (five-leaf stage) were treated with 276 g ai ha^–1^ of tembotrione following the same procedure as described previously. The response of F_2_ plants was assessed by visual injury rating (as described previously) at 2 and 3 WAT (Abit et al., 2009). Further, plants were grouped as highly injured/dead (susceptible) or minor/no symptoms (resistant) at 4 WAT in comparison with the parental genotypes. In addition, total leaf chlorophyll index was estimated in parents and F_2_ progeny on 3 and 4 WAT. Chlorophyll index was measured at three different spots on the leaf blade along the length of the youngest fully opened leaf using a self-calibrating soil plant analysis development (SPAD) chlorophyll meter (Konica Minolta SPAD 502 Chlorophyll Meter, Chiyoda City, Tokyo, Japan). The chlorophyll index obtained from the three spots were averaged and considered as a total leaf chlorophyll index. However, the leaf chlorophyll index was recorded from the second run of S-1 × G-200 F_2_ evaluation which was used for the quantitative trait loci (QTL) mapping experiment (described later in a separate section).

### *HPPD*-Gene Sequencing

The *HPPD* gene from G-200, G-350, and S-1 were sequenced to determine if any target site alterations confer resistance to tembotrione. Leaf tissue (three-leaf stage plants) was collected from three plants (biological replicates) of each genotype grown in the greenhouse as described previously and under similar growth conditions. The genomic DNA was extracted using GeneJET Plant Genomic DNA Purification Mini Kit (Thermo Fisher Scientific, Waltham, MA, United States) following the manufacturer’s instructions. The concentration of the DNA samples was quantified using NanoDrop (Thermo Fisher Scientific). The sorghum *HPPD* gene ∼2 kb was amplified using the primers Sg_HPPD F (5′ GACACGATGAATGCCCATGC 3′) and Sg_HPPD R (5′ AGAGAGATGACAGTACAGTGTTGT 3′) designed from Sobic.002G104200.1 in the sorghum reference genome V3.1.1 (McCormick et al., 2017). PCR was performed using T100 Thermal Cycler (Bio-Rad, Hercules, CA, United States). The PCR mixture contained 50–80 ng of gDNA, 0.5 μM each of forward, reverse primer, and 1 × of GoTaq G2 Green Master Mix (Promega, Madison, WI, United States). PCR amplification was done using the following PCR cycling conditions: initial denaturation at 94°C for 5 min, followed by 35 cycles of denaturation at 94°C for 30 s, annealing at 60°C for 45 s, extension at 72°C for 45 s, and final extension at 72°C for 7 min. The PCR products were analyzed in 1.5% agarose gel to confirm the targeted amplicon size and purified using GeneJET PCR Purification Kit (Thermo Fisher Scientific, Waltham, MA, United States). The PCR purified samples were sequenced by Sanger sequencing service provided by GENEWIZ (South Plainfield, New Jersey, United States). The sequences were aligned using Clustal Omega multiple sequence alignment tool (EMBL-EBI) to check for the mutations.

### CYP-Inhibitor Study

To determine if CYP-mediated metabolism of tembotrione confers resistance in G-200 and G-350 genotypes, experiments were conducted using two CYP inhibitors, malathion and PBO. The sorghum genotypes G-200, G-350, and S-1, along with Pioneer 84G62 and a corn genotype B73, were grown in the greenhouse (as described previously and under similar growth conditions). Malathion (Spectracide malathion insect spray concentrate; Spectrum Brands) at 0, 2,000, and 4,000 g ai ha^–1^ or PBO (Thermo Fisher Scientific, Waltham, MA, United States) at 4,500 g ai ha^–1^ along with 0.25% non-ionic surfactant (NIS) was applied 1 h before tembotrione treatment. Soil drenching of 5 mM malathion 24 h after primary application as a booster dose was given only for the malathion treatments. Tembotrione was applied at 0, 92, 184, and 368 g ai ha^–1^ with 0.25% methylated soy oil. All the treatments were arranged in a factorial design. The same procedure, as mentioned in the earlier tembotrione dose-response assay, was followed for chemical treatments (malathion, PBO, and tembotrione) and data collection.

### Genotyping by Sequencing (GBS)

A total of 150 plants of a single F_2_ family derived from S-1 × G-200 (mentioned as Run-2 in Table 4), along with parents, were grown in the greenhouse as described previously and under the same growth conditions. An equal amount (two 2-cm leaf bits; ∼150 mg) of leaf tissue was collected from all plants in 96-deep-well plates. One 3.2-mm stainless steel bead was added to each well and the leaf tissue was ground for 3 min at 20 cycles per second to obtain fine powder in a Mixer Mill (Retsch GmbH, Haan, North Rhine-Westphalia, Germany). Genomic DNA was extracted using the cetyltrimethylammonium bromide (CTAB) method (Bai et al., 1999) with minor modifications. The DNA concentration in the extracted samples was quantified by FLUOstar Omega microplate reader (BMG LABTECH, Ortenberg, Baden-Württemberg, Germany) using a Quant-iT PicoGreen dsDNA Assay Kit (Life Technologies, Grand Island, NY, United States). Each sample was normalized to contain 10 ng/μl DNA using QIAgility Liquid Handling System (Qiagen, Germantown, MD, United States) for library construction. Approximately 150 ng of genomic DNA of each sample was used to construct a library following the tGBS protocol (Ott et al., 2017) with modifications, and the DNA library was sequenced on the HiseqX 10 platform at Novogene Corporation, Sacramento, CA, United States. Sequencing reads were trimmed and de-barcoded using the pipeline described in the previous tGBS study (Ott et al., 2017). Clean reads of each sample were aligned to *Sorghum bicolor* genome (GenBank accession GCA_000003195.3) (Paterson et al., 2009) using BWA v0.7.12-r1039 (Li and Durbin, 2009) and unique mapped reads were retained for variant discovery using HaplotypeCaller in GATK v4.1 (McKenna et al., 2010). GATK SelectVariants with parameters (-select-type SNP –restrict-alleles-to BIALLELIC -select “QD ≥ 10.0” -select “DP ≥ 200.0”) was applied to filter variants. The SNPs were converted to ABH format (“A” represents resistant parent allele, “B” represents susceptible allele, and “H” represents heterozygous allele) and only polymorphic SNPs between the R and S genotypes were retained using a custom-made Microsoft Excel template. The filtered polymorphic SNPs were used for the construction of a linkage map and QTL analysis.

### Linkage and QTL Mapping

The linkage map was obtained using QTL IciMapping (version 4.5). The grouping and ordering of 606 polymorphic SNP markers were carried out using the regression mapping algorithm RECORD (REcombination Counting and ORDering) based on recombination events between adjacent markers. Further, rippling was done for fine-tuning of the ordered markers on their respective chromosomes by the sum of adjacent recombination fractions (SARF) algorithm with a default window size. The QTL mapping for recovery (RE) and visual injury (VI) was performed using the inclusive composite interval mapping (ICIM) method with additive assumption was performed using the QTL IciMapping (version 4.5) (Meng et al., 2015). The logarithm of the odds (LOD) significance thresholds (*P* < 0.05) were determined by running 1,000 permutations (Churchill and Doerge, 1994). Previously reported QTLs for similar regions were obtained from sorghum QTL Atlas (Mace et al., 2019). The QTLs (q) were named based on the trait abbreviation followed by the chromosome number.

### Statistical Analysis

Dry biomass (% of non-treated) was calculated following the formula:

$$\begin{array}{rc} & \mathrm{Dry biomass}\;(\mathrm{\% of non}-\mathrm{treated})=\\ & \frac{\mathrm{Biomass of individual plant}\;(g)}{\mathrm{Average biomass of the non}-\mathrm{treated plants of the genotype}\;(g)}\\ & \times 100 \end{array}$$

Tembotrione dose-response data expressed as dry biomass (% of non-treated) or percent injury were subjected to non-linear regression analysis using a three- or four-parameter log-logistic model using a “drc” (Ritz and Streibig, 2005) package in R (Development R Core Team, 2013) following Knezevic et al. (2007) and Shyam et al. (2019) to estimate GR_50_ (dose required for 50% growth reduction) or ID_50_ (dose required for 50% visual injury). A “Lack-of-fit” test was performed using the “model fit” function of “drc” to assess the fit of data to various regression models. Differences between the estimated GR_50_ or ID_50_ values were tested with each other by *t*-test using the “compParm” function in the “drc” package. The dose-response curves were generated using the “plot” function in the “drc” package.

ANOVA was performed following Fisher’s LSD test to separate means and significance at *P* ≤ 0.05 using the “agricole” package in R (de Mendiburu, 2014). The plots were generated using the “R” package “ggplot2” (Wickham and Wickham, 2007). A χ^2^ goodness-of-fit test (Cochran, 1952) was used to fit to a single dominant gene by comparing the observed and expected segregation frequencies of tembotrione-resistant or tembotrione-susceptible plants.

## Results

### Identification of Tembotrione-Resistant Genotypes

Among 317 genotypes from the SAP initially screened under *in vitro* (tissue culture) conditions, 10 genotypes showed ≤ 70% tembotrione injury at 2 WAT with significant recovery by 4 WAT (Supplementary Table S1). One genotype, S-1, was found highly susceptible to tembotrione as these plants did not survive tembotrione treatment. In response to the tembotrione application at 92 g ai ha^–1^ (field recommended dose of tembotrione), under greenhouse conditions, out of the above 10 genotypes, only two, i.e., G-200 and G-350, showed the least injury at 2 WAT (73 and 70%, respectively) and the smallest dry biomass reduction at 3 WAT (25.36 and 25.77%, respectively) (Supplementary Table S2). In the treated plants, the tembotrione injury symptoms appeared by 2 WAT, after which in G-200 and G-350, the symptoms dissipated, and plants started to recover. However, leaf chlorosis and bleaching symptoms, typical of tembotrione injury, were visible on all 10 genotypes at a variable degree. The susceptible genotype, S-1, and Pioneer 84G62 (a commercially used sorghum hybrid) exhibited 100 and 90% injury with a biomass reduction of 2.69 and 11.63%, respectively (Supplementary Table S2).

### Tembotrione Dose-Response Assay

**FIGURE 1:**
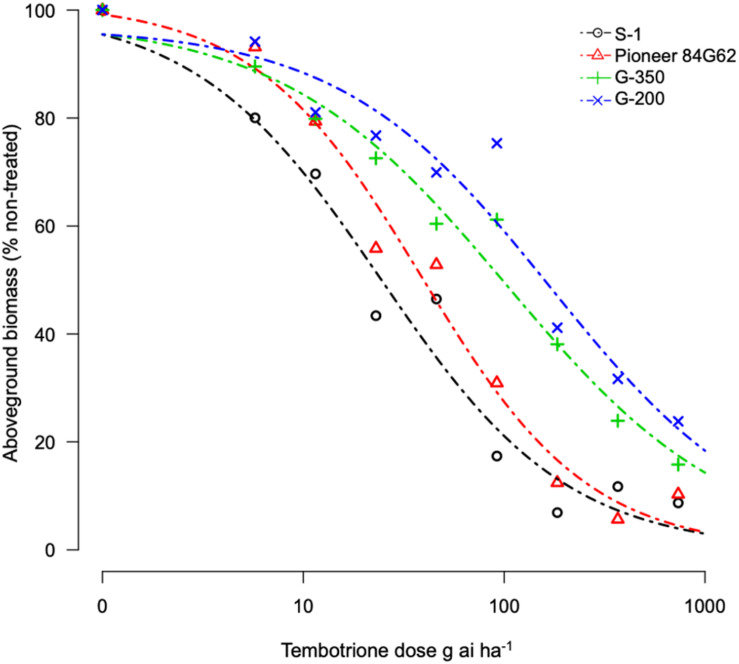
Tembotrione dose-response curves obtained by non-linear regression analysis of above-ground dry biomass of S-1 (susceptible), Pioneer 84G62 (commercial hybrid), G-200, and G-350 (resistant) using the three-parameter log-logistic model.

Based on the GR_50_ values the resistant genotypes, G-200 and G-350 required 215 and 154 g ai ha^–1^ of tembotrione, respectively, for 50% growth reduction which is higher than the field recommended dose of 92 g ai ha^–1^, whereas Pioneer 84G62 and S-1 required only 22 and 36 g ai ha^–1^ for the same level of growth reduction. The GR_50_ values of G-200, G-350, and Pioneer 84G62 were significantly different from S-1. Based on GR_50_ values, compared with S-1, the genotypes G-200, G-350, and Pioneer 84G62 were ∼10 ×, 7 ×, and 1.5 × more resistant, respectively, to tembotrione (Figure 1 and Table 1).

**TABLE 1:** Regression parameters describing the response of sorghum genotypes to tembotrione under greenhouse and field conditions.

Genotype	Greenhouse		Field	
	GR_50_ (SE)	RI (R/S)	ID_50_ (SE)	RI (R/S)
S-1	22.1 (3.4)	1	68.4 (14.3)	1.0
Pioneer 84G62	36.7 (5.6)	2	62.4 (51.0)	1.0
G-350	154.4(68.8)**	7	96.6(17.8)*	1.5
G-200	215.2(160)*	10	129.6(71.8)**	2.0

GR_50_, dose required for 50% growth reduction; ID_50_, dose required for 50% visual injury; SE, standard error; RI, resistance index; R/S, resistant/susceptible. Significantly different from S-1 at * p ≤ 0.05, ** p ≤ 0.01.

### Field Testing

**FIGURE 2:**
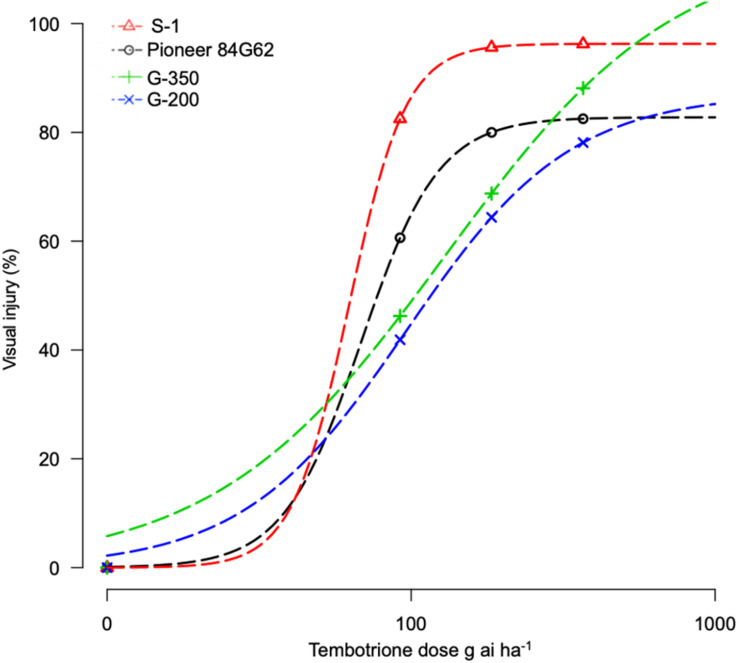
Tembotrione dose-response curves representing the percent injury of S-1 (susceptible), Pioneer 84G62 (commercial hybrid), G-200, and G-350 (resistant genotypes) in response to different doses of tembotrione at 2 weeks after treatment under field conditions.

The response of sorghum genotypes, G-200 and G-350, along with S-1 and Pioneer 84G62, to POST application of tembotrione was tested under field conditions at two sites, Manhattan and Hays, KS. Because site by herbicide dose interaction was non-significant, data for tembotrione injury and yield were pooled and averaged across the two sites. In response to the tembotrione (POST) application, sorghum genotypes showed leaf chlorosis and bleaching symptoms followed by necrotic lesions in susceptible plants. The tembotrione injury was visible on all four sorghum genotypes at 1 WAT. A dose-response curve was generated using the injury ratings at 2 WAT. Based on percent injury at 2 WAT and ID_50_ values, the genotype G-200 required the highest dose (129 g ai ha^–1^) of tembotrione followed by G-350 (96 g ai ha^–1^) genotype. Susceptible genotypes S-1 (62 g ai ha^–1^) and Pioneer 84G62 required the lowest dose of tembotrione (68 g ai ha^–1^) that caused 50% injury (Figure 2 and Table 1). Days required for 50% recovery (ED_50_) from herbicide damage was also calculated. The genotypes, G-200 and G-350, recovered with no injury symptoms by 6 WAT at all doses of tembotrione, whereas Pioneer 84G62 and S-1 took 8 weeks for recovery from 1 × and 2 × of application dose, and S-1 did not survive 4 × dose of tembotrione (Table 2). In response to 1 × dose of tembotrione, no significant reduction in grain yield of G-200 and G-350 was found, whereas the susceptible genotypes, S-1 and Pioneer 84G62, showed ∼40 and 30% grain yield reduction (Table 2) compared with no treatment. However, when treated with 2 × and 4 × doses of tembotrione, grain yields were significantly reduced in all genotypes (Table 2).

**TABLE 2:** Recovery (ED_50_) and single plant yield (% of non-treated) in response to different doses of tembotrione under field conditions (ED_50_: days required for 50% recovery).

Herbicide dose (g ai ha^–1^)	ED_50_ (SE)				Yield (%)			
	S-1	Pioneer 84G62	G-350	G-200	S-1	Pioneer 84G62	G-200	G-350
0	–	–	–	–	100a	100a	100a	100a
92	28 (6)	23 (2)	22 (3)*	18 (1)*	53.7de	68.5cd	95.3ab	99.8a
184	30 (10)	25 (3)	21 (2)*	20 (1)*	19.4g	45.4cd	72.2cd	78.0bc
368	–	27 (1)	21 (3)	19 (1)	–	14.6g	25.3fg	33.3fg

Significantly different from S-1 at * p ≤ 0.05. Numbers with different letters are significantly different; numbers with the same letters are not significantly different.

### Mechanism of Tembotrione Resistance

**FIGURE 3:**
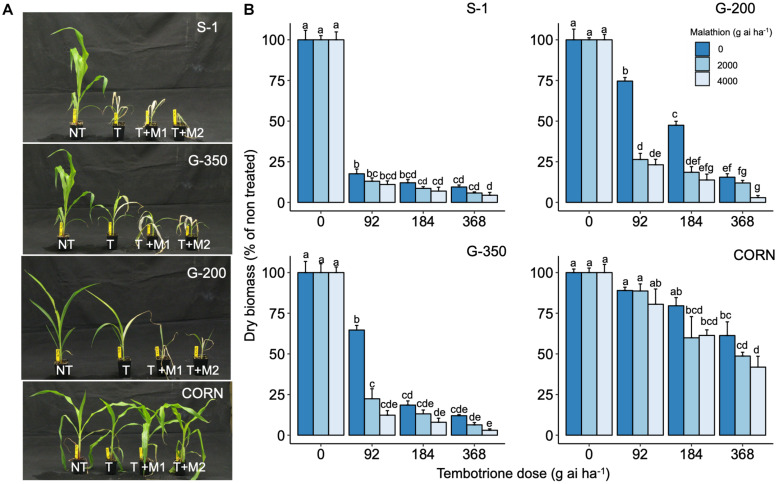
**(A)** The response of S-1 (susceptible), G-200, G-350 (resistant), and corn to field recommended dose tembotrione (92 g ai ha^–1^) (T) or malathion treatments (M1: 2,000 g ai ha^–1^; M2: 4,000 g ai ha^–1^) followed by field recommended dose tembotrione. NT: non-treated. **(B)** Above-ground dry biomass of sorghum genotypes when pre-treated with malathion or different doses of tembotrione. The error bars represent the standard error (*n* = 8); different alphabets indicate a significant difference between treatments (*p* ≤ 0.05).

**FIGURE 4:**
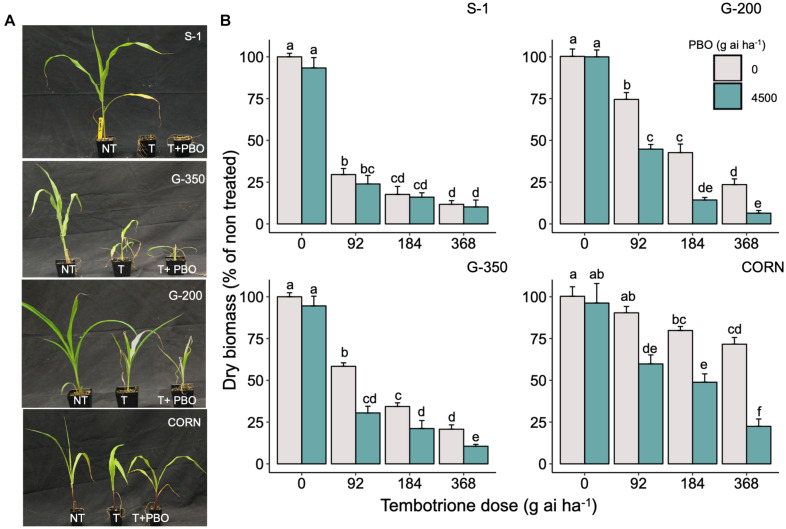
**(A)** The response of S-1 (susceptible), G-200, G-350 (resistant), and corn to field recommended dose of tembotrione (92 g ai ha^–1^) (T) or piperonyl butoxide (PBO) treatment (M1: 4,500 g ai ha^–1^) followed by field recommended dose tembotrione. NT: non-treated. **(B)** Above-ground dry biomass of sorghum genotypes when pre-treated with PBO or different doses of tembotrione. The error bars represent the standard error (*n* = 8); different alphabets indicate a significant difference between treatments (*p* ≤ 0.05).

Upon sequencing the *HPPD* gene from G-200, G-350, and S-1, no mutations were identified in the coding region of the *HPPD* gene (Supplementary Data 1), suggesting that no target site alterations confer resistance to tembotrione in G-200 or G-350. In response to malathion or PBO followed by tembotrione treatments, G-200 and G-350 exhibited significant biomass reduction compared with plants treated with tembotrione alone. Malathion or PBO without tembotrione treatment had no effect on the sorghum genotypes tested (Figures 3, 4). The corn inbred line B73 (known to be resistant to tembotrione) did not show any significant biomass reduction at 92 or 184 g ai ha^–1^ of tembotrione application (Figure 3A); however, it exhibited a significant reduction in biomass in response to pre-treatment with malathion followed by 184 and 368 g ai ha^–1^ of tembotrione (Figure 3B). The genotype G-200 treated with malathion followed by 92, 184, and 368 g ai ha^–1^ showed more than 50% reduction in biomass compared with plants treated only with tembotrione. There is no significant difference in biomass accumulation when treated with 2,000 or 4,000 g ai ha^–1^ of malathion, except in malathion followed by 368 g ai ha^–1^ tembotrione treatment (Figure 3B), whereas G-350 showed significant biomass reduction only when treated with malathion followed by 92 g ai ha^–1^ tembotrione. The susceptible genotype, S-1, showed significant growth reduction at all doses of tembotrione or when pre-treated with malathion (Figure 3B). The corn B73 exhibited significant biomass reduction in response to PBO, followed by 92, 184, and 368 g ai ha^–1^ of tembotrione treatment (Figures 4A,B). Both G-200 and G-350 genotypes also showed a significant reduction in biomass when pre-treated with PBO followed by all doses of tembotrione. The S-1 was susceptible to all treatments applied (Figure 4B).

### Inheritance of Tembotrione Resistance

**FIGURE 5:**
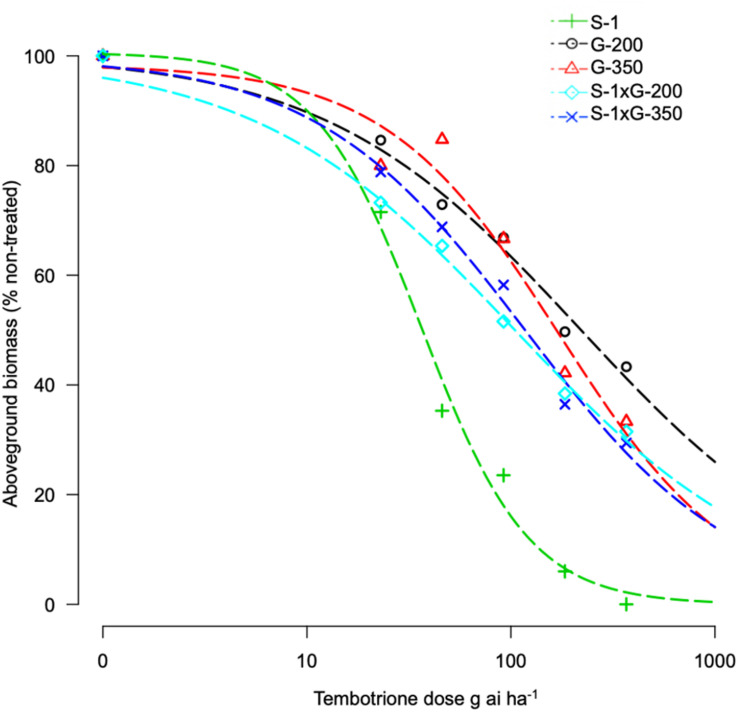
Tembotrione dose-response curves representing the above-ground dry biomass of S-1 (susceptible), F_1_ (S-1 × G-200), F_1_ (S-1 × G-350), and G-200, G-350 (resistant) using the four-parameter log-logistic model.

**FIGURE 6:**
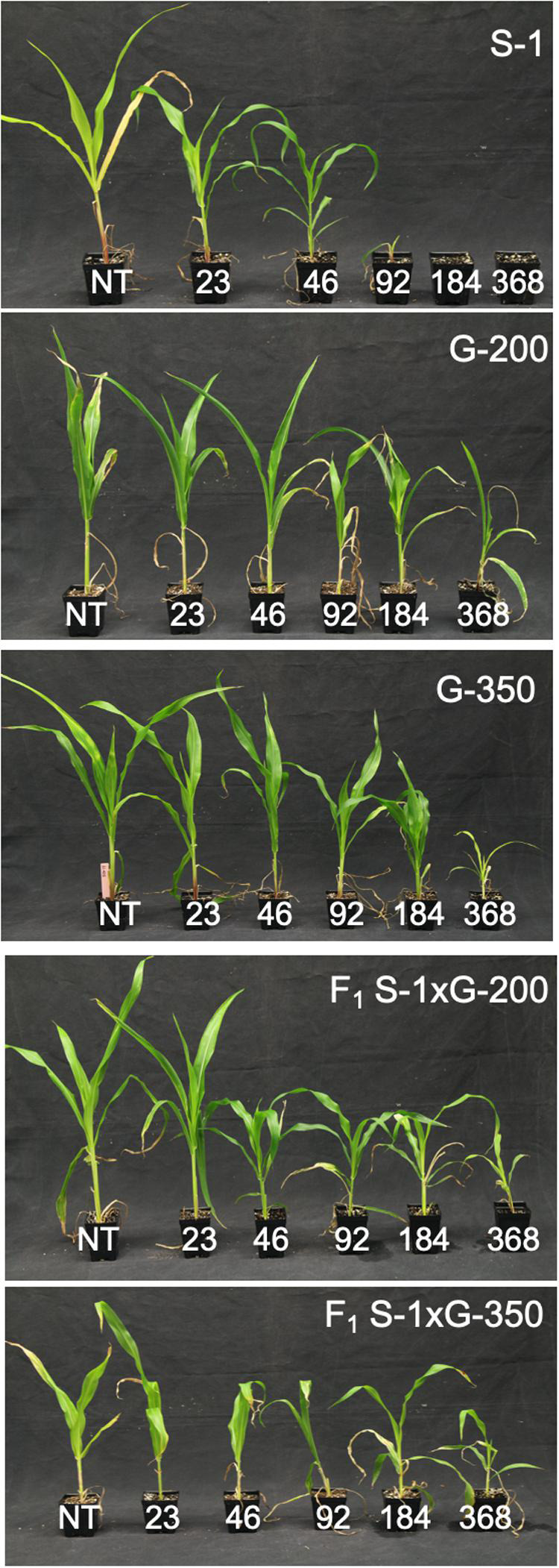
Response of parents S-1 (susceptible), G-200, G-350 (resistant), F_1_ (S-1 × G-200), and F_1_ (S-1 × G-350) to different doses of tembotrione.

The F_1_ progeny of S-1 × G-200 and S-1 × G-350 showed an intermediate response relative to parents when treated with several doses of tembotrione. The GR_50_ of S-1 × G-200 and S-1 × G-350 were estimated at 104 and 117 g ai ha^–1^, respectively, which were less than their respective tembotrione-resistant parents, i.e., G-200 (218 g ai ha^–1^) and G-350 (172 g ai ha^–1^) (Figures 5, 6 and Table 3), suggesting that tembotrione resistance is a partially dominant trait. The F_2_ progeny exhibited a continuous variation for tembotrione injury and recovery. Therefore, to perform a χ^2^-test frequency of segregation of tembotrione resistance or susceptibility in F_2_ progeny, the plants that had more than 80% tembotrione injury were grouped as susceptible and others as resistant. The observed segregation of resistant:susceptible (R:S) ratios from both the crosses did not comply with the expected ratios of 3:1 (R:S) for a single gene inherited trait, indicating that more than one gene is involved in tembotrione resistance in G-200 or G-350 genotypes of sorghum (Table 4).

**TABLE 3:** Regression parameters describing the response of sorghum genotypes and their F_1_ progeny to tembotrione under greenhouse conditions.

Genotype	GR_50_ (SE)	RI (R/S)
S-1	36 (4)	1.0
S-1 × G-200	104(27)**	2.8
S-1 × G-350	117(25)**	3.2
G-350	172(33)**	4.0
G-200	218(60)**	6.0

GR_50_, dose required for 50% growth reduction; SE, standard error; RI, resistance index; R/S, resistant/susceptible. Significantly different from S-1 at ** p ≤ 0.01.

**TABLE 4:** Chi-square analysis of the segregation of tembotrione-resistant (R) and tembotrione-susceptible (S) phenotypes in sorghum F_2_ progeny at 4 weeks after treatment.

Cross	Run	Total	R	S	*P*-value
S-1 × G-200	1	200	168	32	0.0032**
	2	150	124	26	0.0372*
	Runs 1and 2 combined	350	292	58	0.0003**
S-1 × G-350	1	220	197	23	0.0000**

Significantly different at ** p ≤ 0.01, * p ≤ 0.05.

### Mapping Tembotrione Resistance

**FIGURE 7:**
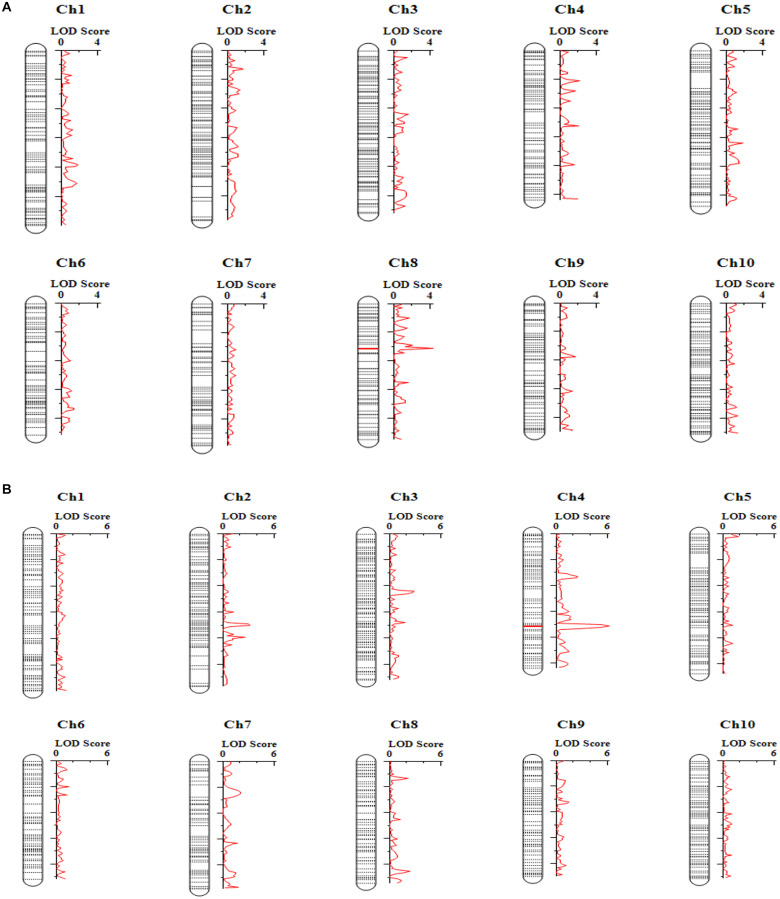
Quantitative trait loci (QTLs) detected from analysis of 150 plants from a single F_2_ family of S-1 × G-200 for different traits: **(A)** recovery (RE); **(B)** visual injury 21 days after treatment (VI).

To map the genomic loci controlling tembotrione resistance, a total of 208,376 SNPs were obtained using GBS from 150 F_2_ progeny (S-1 × G-200) and parents (S-1, G-200). A subset of 1,954 SNP markers polymorphic to both parents with less than 30% missing values were retained. Further, filtering for missing rate (> 90%), strong segregation distortion, marker distribution, and redundant markers resulted in a total of 696 markers that were used for construction of a linkage map. The map of 1,021 cM was prepared which had an average distance of 1.7 cM between two adjacent markers. A total of three QTLs on chromosomes 2, 4, and 8 were mapped with a high LOD score (LOD > 3) (Figure 7 and Table 5) for two traits, RE, i.e., difference between leaf chlorophyll index at 2 and 4 WAT, and visual scoring at 2 WAT VI obtained from 150 F_2_ plants (Supplementary Figure S1). The LOD score of detected QTLs ranged from 3.0 to 6.0 and the phenotypic variations explained (PVE) values ranged from 9 to 44%.

**TABLE 5:** Quantitative trait loci (QTLs) detected for the recovery (RE) and visual injury 3 weeks after treatment (VI) along with logarithm of the odds (LOD) and phenotypic variation explained (PVE) explained by QTLs.

Trait	QTL	Left marker	Right marker	LOD	PVE (%)	Add	Previously reported trait	References
RE	qRE8.1	Ch8_20217087	Ch8_20519857	4.32	44.35	−1.97	Efficiency of PSII reaction centers, chlorophyll fluorescence	Fiedler et al., 2014; Ortiz et al., 2017
VI	qVI4.0	Ch4_46023941	Ch4_48400958	6.20	21.23	17.18	–	–
VI	qVI2.1	Ch2_45821038	Ch2_46847104	3.15	9.71	11.79	–	–

## Discussion

### Identification and Characterization of Tembotrione-Resistant Sorghum

Tembotrione, an HPPD inhibitor, is widely used in corn for POST control of both grasses and broad-leaved weeds, but not registered for use in sorghum due to crop injury (Damalas et al., 2018). In this study, we identified two sorghum genotypes, G-200 and G-350, with a high level of resistance and one genotype S-1 susceptible to tembotrione from SAP. Even though cultivated sorghum hybrids are susceptible to tembotrione, variable levels of responses were reported in sorghum germplasm upon treatment with tembotrione (Cunha et al., 2016). The genotypes, G-200 and G-350, were found ∼10-fold and 7-fold more resistant, respectively, under greenhouse and ∼2-fold more resistant under field conditions as compared with S-1 and Pioneer 84G62 (Table 1). In susceptible genotypes, the application of tembotrione causes foliar bleaching and leaf necrosis followed by complete plant death (Galon et al., 2016). Interaction of genotypes with environmental factors such as rainfall, soil type, and weather conditions plays a key role in plant response to HPPD inhibitors (Bollman et al., 2008), which may explain the difference in the level of resistance between greenhouse and field conditions. Variation in the efficacy of HPPD inhibitors in response to environmental factors such as temperature and relative humidity were reported in several weeds (Johnson and Young, 2002; Godar et al., 2015). However, in both greenhouse and field conditions, G-200 and G-350 survived the field dose of tembotrione, whereas S-1 or Pioneer 84G62 were severely injured (Table 1).

### Mechanism of Tembotrione Resistance

The natural resistance to tembotrione in G-200 and G-350 appears to be not conferred by any alteration to the molecular target of this herbicide, i.e., *HPPD* gene, because no difference in *HPPD* gene sequence was found between the resistant (G-200 and G-300) or susceptible (S-1) genotype (Supplementary Data S1). Likewise, no naturally evolved mutations in the *HPPD* gene that confer resistance to HPPD inhibitors were found in plants (Lu et al., 2020). Nonetheless, recently, soybean varieties resistant to HPPD inhibitors were developed through transgenic technology by inserting an insensitive *HPPD* gene (Siehl et al., 2014; Dreesen et al., 2018).

The CYP enzymes are known to metabolize HPPD inhibitors such as mesotrione (Ma et al., 2013; Nakka et al., 2017), tembotrione (Küpper et al., 2018), or topramezone (Elmore et al., 2015) in naturally evolved resistant weed biotypes. In this research, the genotypes G-200 and G-350 exhibited significant biomass reduction in response to pre-treatment with CYP inhibitors, malathion, or PBO followed by tembotrione application, suggesting that tembotrione is metabolized by CYP activity (Figures 3, 4). Similarly, the use of these inhibitors, followed by tembotrione, showed ∼10% more biomass reduction compared with tembotrione alone treatments in a tembotrione-resistant common waterhemp population (Oliveira et al., 2018). Metabolism of tembotrione by hydroxylation followed by glycosylation, catalyzed by CYPs, has also been reported in a tembotrione-resistant Palmer amaranth biotype (Küpper et al., 2018). Furthermore, RNA-Seq analysis revealed differential expression of several CYP genes, for example, three to fourfold upregulation of *CYP72A219* and *CYP81E8*, respectively, was found in the same aforementioned tembotrione-resistant Palmer amaranth biotype (Küpper, 2018). In corn, multiple CYPs located in *nsf1* locus were found to metabolize tembotrione, mesotrione, as well as other herbicides (Williams and Pataky, 2010).

### Inheritance of Tembotrione Resistance in Sorghum

Based on the response of F_1_ progeny (S-1 × G-200 and S-1 × G-350) to tembotrione treatment, we found that the tembotrione resistance in G-200 and G-350 is a partially dominant trait (Figures 5, 6 and Table 3). Furthermore, F_2_ data demonstrated that this resistance is controlled by multiple genes (Table 4). Genetic analyses of sweet corn inbred lines revealed a single recessive allele controlling the sensitivity to tembotrione (Williams and Pataky, 2008). The genetic basis of tembotrione resistance is not extensively studied in plants; however, mesotrione (another widely used HPPD inhibitor) resistance in several common waterhemp populations across US Midwest (Huffman et al., 2015; Kohlhase et al., 2018; Oliveira et al., 2018) was found to be inherited by a partially dominant polygenic trait.

### QTL Mapping

We mapped three QTLs associated with tembotrione resistance on chromosomes 2, 4, and 8 using the sequence data from 150 F_2_ plants from S-1 × G-200 cross (Figure 7). To our knowledge, this is the first report of QTLs associated with tembotrione resistance in grain sorghum. The QTL mapped using RE were previously reported for other traits in sorghum related to chlorophyll fluorescence (Fiedler et al., 2014) and photochemical quenching (Ortiz et al., 2017; Table 5); the QTLs mapped on chromosomes 2 and 4 using VI were novel QTLs and not previously reported for any other trait. The QTLs need to be tested in multiple environments with more number of F_2_ plants and markers to improve the estimation accuracy, and experiments are in progress to further fine map and identify the precise location of the gene(s) responsible for tembotrione resistance in grain sorghum.

As mentioned earlier, our data indicate that the tembotrione resistance is a polygenic trait, and such traits can express differently in different genetic backgrounds. Therefore, tembotrione resistance can potentially be improved by crossing G-200 and G-350 or with other commercial genetic backgrounds. Such work has been reported to enhance the performance of quantitative traits in different genetic backgrounds and environmental conditions such as drought (Reddy et al., 2009), stay green (Subudhi et al., 2000), cold tolerance (Knoll and Ejeta, 2008), and yield (Nagaraja-Reddy et al., 2013) in grain sorghum. Therefore, there is enormous potential for improving tembotrione resistance by testing the expression of this trait in different genetic backgrounds and for the development of tembotrione-resistant sorghum varieties.

Because sorghum can outcross with closely related wild and weedy species, such as johnsongrass or shattercane, one of the major concerns of the development of herbicide-resistant sorghum varieties has been a natural transfer of such resistance into these weed species (Ohadi et al., 2017). However, recent reports suggest that the outcrossing rate of sorghum with johnsongrass was as low as ∼1% under controlled conditions (Hodnett et al., 2019) and 2–16% with shattercane under field conditions (Schmidt et al., 2013). Although the possibility of outcrossing is minimal, if an herbicide resistance trait escapes into the wild species, necessary stewardship practices must be developed and integrated into sorghum weed management practices.

In conclusion, we have identified sorghum genotypes (G-200 and G-350) with natural resistance to tembotrione from the SAP, which can potentially be used to introgress the tembotrione resistance into breeding lines by conventional or marker-assisted breeding methods. CYP-inhibitor assay suggested CYP-mediated metabolism of tembotrione in the resistant genotypes. Genetic analyses of F_1_ and F_2_ progeny demonstrated that the resistance is a partially dominant polygenic trait. Furthermore, GBS-based QTL mapping revealed three QTLs associated with tembotrione resistance in grain sorghum. Future research needs to be focused on incorporating the resistant trait with elite breeding varieties, testing the hybrid performance, and improving herbicide resistance in high yielding and stress tolerance hybrids.

## Data Availability Statement

The datasets generated for this study can be found in NCBI (BioProject ID: PRJNA657005).

## Author Contributions

BP performed the inheritance experiments, CYP-inhibitor assays, QTL mapping, curated the data, and wrote the first draft of the manuscript. AV performed *in vitro* and greenhouse experiments. BP, AV, and ARV conducted dose-response experiments and field testing. MT assisted high-throughput DNA extraction. MZ prepared the GBS library. SL, RS, GL, and BP performed the QTL mapping. TT provided the seed material and input on genetic analyses. MJ and PVVP designed and conceived the experiments, analyzed the data, and helped to draft the final manuscript. All authors read, edited, and approved the final version of the manuscript.

## Conflict of Interest

The authors declare that the research was conducted in the absence of any commercial or financial relationships that could be construed as a potential conflict of interest.
