# Supplementary material for: Characterization, Genetic Analyses, and Identification of QTLs Conferring Metabolic Resistance to a 4-Hydroxyphenylpyruvate Dioxygenase Inhibitor in Sorghum (Sorghum bicolor)
Source: Front Plant Sci. 2020 Dec 9;11:596581. doi: 10.3389/fpls.2020.596581 (PMC7756693; doi:10.3389/fpls.2020.596581)
Supplement: Supplementary file 1 [file Table_1.DOCX]

**DATA S1.** Sequence alignment of 4-Hydroxyphenylpyruvate dioxygenase (*HPPD*) gene from resistant genotypes G-200, G-350, and susceptible genotype S-1.

Sobic.002G104200.1 ATGAATGCCCATGCCCACCCAGTTGTGGCCTCCACGGCGTTCGCCCATGCCCTCCTGCCA 60

G-200 ATGAATGCCCATGCCCACCCAGTTGTGGCCTCCACGGCGTTCGCCCATGCCCTCCTGCCA 60

G-350 ATGAATGCCCATGCCCACCCAGTTGTGGCCTCCACGGCGTTCGCCCATGCCCTCCTGCCA 60

S-1 ATGAATGCCCATGCCCACCCAGTTGTGGCCTCCACGGCGTTCGCCCATGCCCTCCTGCCA 60

************************************************************

Sobic.002G104200.1 AACTCCAGCAGCATTCGATCACGACGCACCCCACTCCGCCCAATTTTTGCAGTGCCGCCG 120

G-200 AACTCCAGCAGCATTCGATCACGACGCACCCCACTCCGCCCAATTTTTGCAGTGCCGCCG 120

G-350 AACTCCAGCAGCATTCGATCACGACGCACCCCACTCCGCCCAATTTTTGCAGTGCCGCCG 120

S-1 AACTCCAGCAGCATTCGATCACGACGCACCCCACTCCGCCCAATTTTTGCAGTGCCGCCG 120

************************************************************

Sobic.002G104200.1 CCAGCCCTTGGACAACTCCCGACGACTCTCCGAGCAACAACCAAGACGCTTCTGCACCCA 180

G-200 CCAGCCCTTGGACAACTCCCGACGACTCTCCGAGCAACAACCAAGACGCTTCTGCACCCA 180

G-350 CCAGCCCTTGGACAACTCCCGACGACTCTCCGAGCAACAACCAAGACGCTTCTGCACCCA 180

S-1 CCAGCCCTTGGACAACTCCCGACGACTCTCCGAGCAACAACCAAGACGCTTCTGCACCCA 180

************************************************************

Sobic.002G104200.1 ACGACGACGCCGCCCGTCGTCACCACCACCGCTGACCGTGCTGAAAGCGTTTTCGCCGGC 240

G-200 ACGACGACGCCGCCCGTCGTCACCACCACCGCTGACCGTGCTGAAAGCGTTTTCGCCGGC 240

G-350 ACGACGACGCCGCCCGTCGTCACCACCACCGCTGACCGTGCTGAAAGCGTTTTCGCCGGC 240

S-1 ACGACGACGCCGCCCGTCGTCACCACCACCGCTGACCGTGCTGAAAGCGTTTTCGCCGGC 240

************************************************************

Sobic.002G104200.1 ACCGGCGACCGCTTCCACGTGATGGACTTCCACCACGTCGAGTTCTGGTGCGCCGACGCC 300

G-200 ACCGGCGACCGCTTCCACGTGATGGACTTCCACCACGTCGAGTTCTGGTGCGCCGACGCC 300

G-350 ACCGGCGACCGCTTCCACGTGATGGACTTCCACCACGTCGAGTTCTGGTGCGCCGACGCC 300

S-1 ACCGGCGACCGCTTCCACGTGATGGACTTCCACCACGTCGAGTTCTGGTGCGCCGACGCC 300

************************************************************

Sobic.002G104200.1 GCCTCCGCTGCCGGCCGCTTCTCCTTCGCGCTCGGCGTGCCACTCGCCGCGCAGTCCGAC 360

G-200 GCCTCCGCTGCCGGCCGCTTCTCCTTCGCGCTCGGCGTGCCACTCGCCGCGCAGTCCGAC 360

G-350 GCCTCCGCTGCCGGCCGCTTCTCCTTCGCGCTCGGCGTGCCACTCGCCGCGCAGTCCGAC 360

S-1 GCCTCCGCTGCCGGCCGCTTCTCCTTCGCGCTCGGCGTGCCACTCGCCGCGCAGTCCGAC 360

************************************************************

Sobic.002G104200.1 CTCACCACGGGGAACACCGCGCACGCTTCCCGCCTGCTGCGGTCGCGCTCTGGACCTCTC 420

G-200 CTCACCACGGGGAACACCGCGCACGCTTCCCGCCTGCTGCGGTCGCGCTCTGGACCTCTC 420

G-350 CTCACCACGGGGAACACCGCGCACGCTTCCCGCCTGCTGCGGTCGCGCTCTGGACCTCTC 420

S-1 CTCACCACGGGGAACACCGCGCACGCTTCCCGCCTGCTGCGGTCGCGCTCTGGACCTCTC 420

************************************************************

Sobic.002G104200.1 GCGCTCCTCTTCACCGCCCCGTACGCGCGGCACATCGGCGCCCGCCCCGACGCCGACGCA 480

G-200 GCGCTCCTCTTCACCGCCCCGTACGCGCGGCACATCGGCGCCCGCCCCGACGCCGACGCA 480

G-350 GCGCTCCTCTTCACCGCCCCGTACGCGCGGCACATCGGCGCCCGCCCCGACGCCGACGCA 480

S-1 GCGCTCCTCTTCACCGCCCCGTACGCGCGGCACATCGGCGCCCGCCCCGACGCCGACGCA 480

************************************************************

Sobic.002G104200.1 ACGTCCGCCTCCTCCCCCGTGGTGCCCTCCTTCTCGGCCGACGCCGCGCGCCGCTTCGCC 540

G-200 ACGTCCGCCTCCTCCCCCGTGGTGCCCTCCTTCTCGGCCGACGCCGCGCGCCGCTTCGCC 540

G-350 ACGTCCGCCTCCTCCCCCGTGGTGCCCTCCTTCTCGGCCGACGCCGCGCGCCGCTTCGCC 540

S-1 ACGTCCGCCTCCTCCCCCGTGGTGCCCTCCTTCTCGGCCGACGCCGCGCGCCGCTTCGCC 540

************************************************************

Sobic.002G104200.1 GCCGACTACGGCGGCCTCGCGGTGCGCGCCGTCGCGGTCCGTGTCTCCGACGCCGCCGAG 600

G-200 GCCGACTACGGCGGCCTCGCGGTGCGCGCCGTCGCGGTCCGTGTCTCCGACGCCGCCGAG 600

G-350 GCCGACTACGGCGGCCTCGCGGTGCGCGCCGTCGCGGTCCGTGTCTCCGACGCCGCCGAG 600

S-1 GCCGACTACGGCGGCCTCGCGGTGCGCGCCGTCGCGGTCCGTGTCTCCGACGCCGCCGAG 600

************************************************************

Sobic.002G104200.1 GCGTTCCGCGCCAGCGTCGCCGCGGGTGCGCGCCCGGCCTTCGCTCCCGCTGAGCTCGGC 660

G-200 GCGTTCCGCGCCAGCGTCGCCGCGGGTGCGCGCCCGGCCTTCGCTCCCGCTGAGCTCGGC 660

G-350 GCGTTCCGCGCCAGCGTCGCCGCGGGTGCGCGCCCGGCCTTCGCTCCCGCTGAGCTCGGC 660

S-1 GCGTTCCGCGCCAGCGTCGCCGCGGGTGCGCGCCCGGCCTTCGCTCCCGCTGAGCTCGGC 660

************************************************************

Sobic.002G104200.1 CACGGCTTCGTGTTTGCCGAAGTCGAGCTCTACGGAGACGCCGTCCTCCGTTTCGTGAGC 720

G-200 CACGGCTTCGTGTTTGCCGAAGTCGAGCTCTACGGAGACGCCGTCCTCCGTTTCGTGAGC 720

G-350 CACGGCTTCGTGTTTGCCGAAGTCGAGCTCTACGGAGACGCCGTCCTCCGTTTCGTGAGC 720

S-1 CACGGCTTCGTGTTTGCCGAAGTCGAGCTCTACGGAGACGCCGTCCTCCGTTTCGTGAGC 720

************************************************************

Sobic.002G104200.1 TACCCGGACGACACGGGCGGCGTGGCCTTCCTCCCCGGGTTCGAGAACGTCGCAAACTCA 780

G-200 TACCCGGACGACACGGGCGGCGTGGCCTTCCTCCCCGGGTTCGAGAACGTCGCAAACTCA 780

G-350 TACCCGGACGACACGGGCGGCGTGGCCTTCCTCCCCGGGTTCGAGAACGTCGCAAACTCA 780

S-1 TACCCGGACGACACGGGCGGCGTGGCCTTCCTCCCCGGGTTCGAGAACGTCGCAAACTCA 780

************************************************************

Sobic.002G104200.1 TCAGCGTGCCCGGCGCCGGACTACGGACTCAACCGGTTCGACCACATCGTCGGCGGCGTG 840

G-200 TCAGCGTGCCCGGCGCCGGACTACGGACTCAACCGGTTCGACCACATCGTCGGCGGCGTG 840

G-350 TCAGCGTGCCCGGCGCCGGACTACGGACTCAACCGGTTCGACCACATCGTCGGCGGCGTG 840

S-1 TCAGCGTGCCCGGCGCCGGACTACGGACTCAACCGGTTCGACCACATCGTCGGCGGCGTG 840

************************************************************

Sobic.002G104200.1 CCGGACCTGGCTCCGGTCGCCGCGTACATCGCCGGCTTCACGGGCTTCCACGAATTCGAC 900

G-200 CCGGACCTGGCTCCGGTCGCCGCGTACATCGCCGGCTTCACGGGCTTCCACGAATTCGAC 900

G-350 CCGGACCTGGCTCCGGTCGCCGCGTACATCGCCGGCTTCACGGGCTTCCACGAATTCGAC 900

S-1 CCGGACCTGGCTCCGGTCGCCGCGTACATCGCCGGCTTCACGGGCTTCCACGAATTCGAC 900

************************************************************

Sobic.002G104200.1 AGGGTCAACGGCGACGAAATAGGCACGGCCGAGAGCTCGCTCAACGGCCTGGTGCTGGCG 960

G-200 AGGGTCAACGGCGACGAAATAGGCACGGCCGAGAGCTCGCTCAACGGCCTGGTGCTGGCG 960

G-350 AGGGTCAACGGCGACGAAATAGGCACGGCCGAGAGCTCGCTCAACGGCCTGGTGCTGGCG 960

S-1 AGGGTCAACGGCGACGAAATAGGCACGGCCGAGAGCTCGCTCAACGGCCTGGTGCTGGCG 960

************************************************************

Sobic.002G104200.1 GACAGCTCGGAGAAGGTGCTCCTCACGCTGCTGGAGCCGGTGCAGGGCACCAAGCGCCGG 1020

G-200 GACAGCTCGGAGAAGGTGCTCCTCACGCTGCTGGAGCCGGTGCAGGGCACCAAGCGCCGG 1020

G-350 GACAGCTCGGAGAAGGTGCTCCTCACGCTGCTGGAGCCGGTGCAGGGCACCAAGCGCCGG 1020

S-1 GACAGCTCGGAGAAGGTGCTCCTCACGCTGCTGGAGCCGGTGCAGGGCACCAAGCGCCGG 1020

************************************************************

Sobic.002G104200.1 AGCCAGATACAGACGTTCCTGGACCACCATGGCGGGCCAGGAGTGCAGCACCTGGCCATG 1080

G-200 AGCCAGATACAGACGTTCCTGGACCACCATGGCGGGCCAGGAGTGCAGCACCTGGCCATG 1080

G-350 AGCCAGATACAGACGTTCCTGGACCACCATGGCGGGCCAGGAGTGCAGCACCTGGCCATG 1080

S-1 AGCCAGATACAGACGTTCCTGGACCACCATGGCGGGCCAGGAGTGCAGCACCTGGCCATG 1080

************************************************************

Sobic.002G104200.1 ACCAGTGACGACCTTCTCGGCACGCTGAGGGAGATACGTGCGCGGTCCTCCATGGGCGGC 1140

G-200 ACCAGTGACGACCTTCTCGGCACGCTGAGGGAGATACGTGCGCGGTCCTCCATGGGCGGC 1140

G-350 ACCAGTGACGACCTTCTCGGCACGCTGAGGGAGATACGTGCGCGGTCCTCCATGGGCGGC 1140

S-1 ACCAGTGACGACCTTCTCGGCACGCTGAGGGAGATACGTGCGCGGTCCTCCATGGGCGGC 1140

************************************************************

Sobic.002G104200.1 TTCGAGCTCCTGCCACCGCCGCCGCCCAGCTACTATGACGGCGTAAAGCGGCTCGCCGGG 1200

G-200 TTCGAGCTCCTGCCACCGCCGCCGCCCAGCTACTATGACGGCGTAAAGCGGCTCGCCGGG 1200

G-350 TTCGAGCTCCTGCCACCGCCGCCGCCCAGCTACTATGACGGCGTAAAGCGGCTCGCCGGG 1200

S-1 TTCGAGCTCCTGCCACCGCCGCCGCCCAGCTACTATGACGGCGTAAAGCGGCTCGCCGGG 1200

************************************************************

Sobic.002G104200.1 GATGTGCTGTCGGAGGCGCAGATTAACGAGTGCCAAGAGCTCGGCGTGCGGGTGGACAGG 1260

G-200 GATGTGCTGTCGGAGGCGCAGATTAACGAGTGCCAAGAGCTCGGCGTGCGGGTGGACAGG 1260

G-350 GATGTGCTGTCGGAGGCGCAGATTAACGAGTGCCAAGAGCTCGGCGTGCGGGTGGACAGG 1260

S-1 GATGTGCTGTCGGAGGCGCAGATTAACGAGTGCCAAGAGCTCGGCGTGCGGGTGGACAGG 1260

************************************************************

Sobic.002G104200.1 GCTGACAATGGCGGAGTTGTGCTCCAAACCTTCACCAAGGCTGCTGGAGACAGGCCAACC 1320

G-200 GCTGACAATGGCGGAGTTGTGCTCCAAACCTTCACCAAGGCTGCTGGAGACAGGCCAACC 1320

G-350 GCTGACAATGGCGGAGTTGTGCTCCAAACCTTCACCAAGGCTGCTGGAGACAGGCCAACC 1320

S-1 GCTGACAATGGCGGAGTTGTGCTCCAAACCTTCACCAAGGCTGCTGGAGACAGGCCAACC 1320

************************************************************

Sobic.002G104200.1 TTGCTCTTGGAGTTTATCCAGAGGATCGGCTGCGTGGAGATAGATGAGAACGGGAAGGAA 1380

G-200 TTGCTCTTGGAGTTTATCCAGAGGATCGGCTGCGTGGAGATAGATGAGAACGGGAAGGAA 1380

G-350 TTGCTCTTGGAGTTTATCCAGAGGATCGGCTGCGTGGAGATAGATGAGAACGGGAAGGAA 1380

S-1 TTGCTCTTGGAGTTTATCCAGAGGATCGGCTGCGTGGAGATAGATGAGAACGGGAAGGAA 1380

************************************************************

Sobic.002G104200.1 TACCAGAGGGGTGGCTGCGGCGGTTTTGCCAAGGATAACGTCATTCATTTGGTGAAATCC 1440

G-200 TACCAGAGGGGTGGCTGCGGCGGTTTTGCCAAGGATAACGTCATTCATTTGGTGAAATCC 1440

G-350 TACCAGAGGGGTGGCTGCGGCGGTTTTGCCAAGGATAACGTCATTCATTTGGTGAAATCC 1440

S-1 TACCAGAGGGGTGGCTGCGGCGGTTTTGCCAAGGATAACGTCATTCATTTGGTGAAATCC 1440

************************************************************

Sobic.002G104200.1 ATTGAGGACTATGACAAAACTCTTGACGCTCCTGCCCATGTGGCTTCCTAA 1491

G-200 ATTGAGGACTATGACAAAACTCTTGACGCTCCTGCCCATGTGGCTTCCTAA 1491

G-350 ATTGAGGACTATGACAAAACTCTTGACGCTCCTGCCCATGTGGCTTCCTAA 1491

S-1 ATTGAGGACTATGACAAAACTCTTGACGCTCCTGCCCATGTGGCTTCCTAA 1491
