# Supplementary material for: Characterization, Genetic Analyses, and Identification of QTLs Conferring Metabolic Resistance to a 4-Hydroxyphenylpyruvate Dioxygenase Inhibitor in Sorghum (Sorghum bicolor)
Source: Front Plant Sci. 2020 Dec 9;11:596581. doi: 10.3389/fpls.2020.596581 (PMC7756693; doi:10.3389/fpls.2020.596581)
Supplement: Supplementary file 2 [file Table_2.docx]

**FIGURE S1.** Phenotypic distribution of A) S-1 x G-200 F_2_ progeny and B) parents for Recovery (RE); and Visual injury at four weeks after treatment (VI).

G-200 S-1

G-200 S-1

A)

B)
