# Supplementary material for: Characterization, Genetic Analyses, and Identification of QTLs Conferring Metabolic Resistance to a 4-Hydroxyphenylpyruvate Dioxygenase Inhibitor in Sorghum (Sorghum bicolor)
Source: Front Plant Sci. 2020 Dec 9;11:596581. doi: 10.3389/fpls.2020.596581 (PMC7756693; doi:10.3389/fpls.2020.596581)
Supplement: Supplementary file 3 [file Table_3.DOCX]

**SUPPLEMENTARY FIGURE S2.** Linkage map obtained from 606 markers spread across sorghum genome, Genetic distances are indicated on the left side of linkage group in centiMorgans (cM), and the marker names are shown on the right side.


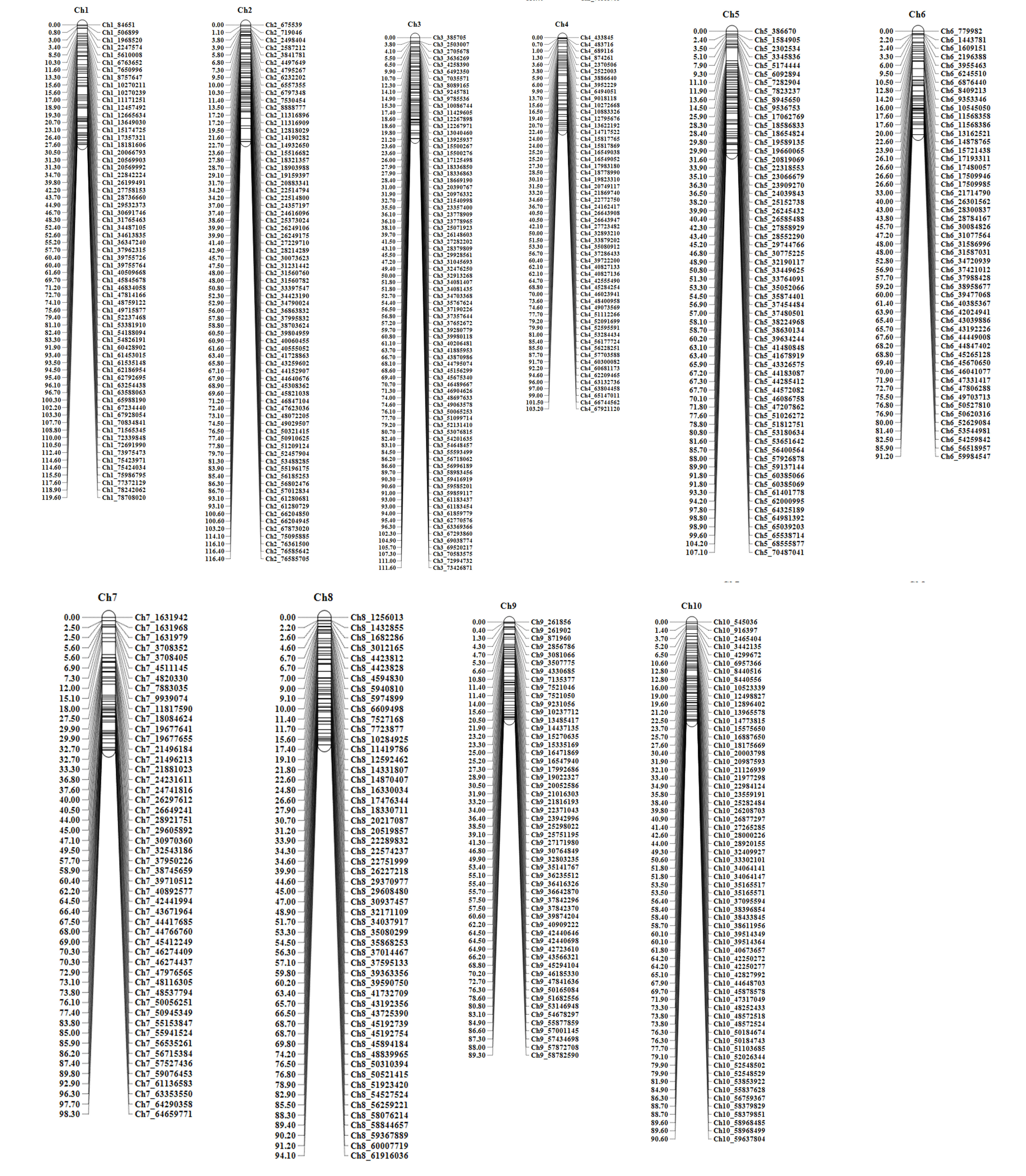
.
