# Supplementary material for: Characterization, Genetic Analyses, and Identification of QTLs Conferring Metabolic Resistance to a 4-Hydroxyphenylpyruvate Dioxygenase Inhibitor in Sorghum (Sorghum bicolor)
Source: Front Plant Sci. 2020 Dec 9;11:596581. doi: 10.3389/fpls.2020.596581 (PMC7756693; doi:10.3389/fpls.2020.596581)
Supplement: Supplementary file 4 [file Table_4.DOCX]

**SUPPLEMENTARY TABLE S1.** Percent injury in selected sorghum association panel genotypes screened on agar media (*in vitro*) containing 0.25 μM of tembotrione.

| Genotype | Injury (%) | |
| --- | --- | --- |
|  | 2 WAT (±SE)^1^ | 4 WAT (±SE) |
| S-1 | 97±1 | 100±0 |
| G-337 | 65±2 | 66±2 |
| G-328 | 50±2 | 59±1 |
| G-404 | 53±1 | 56±1 |
| G- 127 | 48±4 | 55±3 |
| G-196 | 56±1 | 54±2 |
| G-224 | 50±0 | 51±1 |
| G-10 | 51±1 | 50±0 |
| G-1 | 49±1 | 45±0 |
| G-350 | 48±1 | 44±2 |
| G-200 | 48±3 | 41±3 |

^1^WAT, weeks after treatment; SE, standard error
