# Supplementary material for: Characterization, Genetic Analyses, and Identification of QTLs Conferring Metabolic Resistance to a 4-Hydroxyphenylpyruvate Dioxygenase Inhibitor in Sorghum (Sorghum bicolor)
Source: Front Plant Sci. 2020 Dec 9;11:596581. doi: 10.3389/fpls.2020.596581 (PMC7756693; doi:10.3389/fpls.2020.596581)
Supplement: Supplementary file 5 [file Table_5.DOCX]

**SUPPLEMENTARY TABLE S2.** Percent injury (2 weeks after treatment, WAT) and aboveground dry biomass (% untreated, 3 WAT) of selected sorghum association panel genotypes treated with 46 g ai ha^-1^ of tembotrione under greenhouse conditions. All plants were treated at 4 to 5 leaf stage.

| Genotype | Injury (%) | Aboveground dry biomass (%) |
| --- | --- | --- |
| S-1 | 100 | 02.69 |
| Pioneer 84G62 | 90 | 11.63 |
| G-1 | 85 | 11.16 |
| G-224 | 85 | 12.78 |
| G-404 | 85 | 14.47 |
| G-10 | 85 | 16.27 |
| G-127 | 83 | 16.71 |
| G-196 | 82 | 22.98 |
| G-337 | 80 | 23.00 |
| G-350 | 73 | 25.36 |
| G-328 | 82 | 23.49 |
| G-200 | 70 | 25.77 |
